# Desk Rejection Decisions – Do Co-Editors-In-Chief of This Journal Agree?

**DOI:** 10.3389/ijph.2026.1608909

**Published:** 2026-02-26

**Authors:** Nino Künzli, Olaf von dem Knesebeck, Andrea Madarasova Geckova, Sunghea Park, Christopher Woodrow

**Affiliations:** 1 Swiss School of Public Health (SSPH+), Zurich, Switzerland; 2 Swiss Tropical and Public Health Institute, Allschwil, Switzerland; 3 University of Basel, Basel, Switzerland; 4 Institute of Medical Sociology, University of Hamburg, Hamburg, Germany; 5 Department of Health Psychology and Research Methodology, Pavol Jozef Šafárik University in Košice, Košice, Slovakia

**Keywords:** desk rejection, novelty criteria, peer review, rejection rates, scientific publishing

## Abstract

**Objectives:**

Given the growing demand for peer reviews, many public health journals face increasing reluctance from scientists to act as reviewers. The decisions made by pre-screening editors about whether to desk reject a submitted manuscript or initiate peer review are therefore of the utmost importance. The lower the specificity of this decision, the higher the post-peer-review rejection rate, increasing the “rejection cascade” of repeated submissions and peer review cycles. We conducted a two-stage comparison to understand the agreement of pre-screen decisions among the three Co-Editors-in-Chief of the International Journal of Public Health (IJPH), an independent journal of the Swiss School of Public Health (SSPH+).

**Methods:**

In total, the three Co-editors in chief made pre-screen decisions independently (stage 1) and then again after considering others’ views (stage 2).

**Results:**

Full Stage 1 agreement was observed for only 43% of the 30 manuscripts considered. Taking second opinions into account resulted in 67% agreement at stage 2. The main drivers of disagreement were the “soft” criteria that guide the pre-screen decisions, such as “novelty” and “originality”. Stage 1 pre-screen rejection rates of 47%, 80% and 60% for the three editors increased to 57%, 83% and 67% respectively at stage 2.

**Conclusion:**

Based on these findings, IJPH editors will add a “second opinion” for manuscripts they are considering for peer review.

## Introduction

The quality of publications accepted in public health science journals is largely determined by the authors and the collaborative work of the Editors-in-Chief (EiC), the handling editors (HE) who lead the peer review, and the scientists who provide critical reviews. Thus, as also highlighted in the Guidelines of the International Committee of Medical Journal Editors (updated 2024), the scientific competences and expertise of these constituencies is instrumental for the steering of fair, rigorous and reliable peer reviews [1]. Peer review in public health sciences is currently in crisis [2]. In the case of the International Journal of Public Health (IJPH), one of the two independent society journals of the non-profit Swiss School of Public Health (SSPH+) foundation, HEs are challenged by the fact that less than 5% of scientists accept invitations for peer review. Some 5 years ago, until the pandemic, acceptance rates were about 35%. We consider robust peer review, led by active and independent experts in the field, essential to maintain the quality of public health sciences. Finding solutions to the review crisis is therefore of the utmost importance.

As the three Co-EiCs and the independent Editorial Office of the International Journal of Public Health (IJPH), the authors of this piece have a leading responsibility for an efficient, reliable and valid peer review. Thus, understanding the causes of the peer review crisis and our potential roles in finding solutions has become a central interest in our editorial work. Let us highlight four issues that increase the demand for reviews. In turn, these might stress, frustrate and ultimately demotivate scientists who are invited for reviews.

**FIGURE 1:**
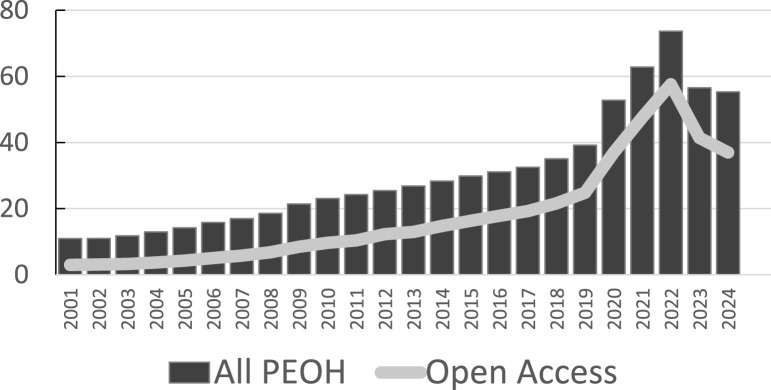
Number of original articles or reviews published in English in the Web-of-Science (WoS) journal category “Public Environmental and Occupational Health” (PEOH), in Thousands, per year (2001–2024) based on Clarivate WoS accessed 15 of May 2025. Bars show the total numbers; the gray line shows the number of publications published open access (OA, all forms). OA increased from 27% to 78% (2021) and declined since to 67% (2024).

First, as shown by Bornmann et al, continuously growing investments in science are paralleled by a steady increase in the number of publications [3], and the number of life science publications increased by approximately 5% per year over the last century. For publications in the Web-of-Science (WoS) category “Public Environmental and Occupational Health” (PEOH), the annual increase since 2000 corresponds to 7% if the peak during the pandemic is ignored. Related data are shown in Figure 1, derived from the Clarivate Web-of-Science website, accessed 25.5.25. If the number of experienced scientists available for peer review does not grow at the same rate, experts will see an increasing demand for reviews. In the case of IJPH, the acceptance rate of peer review invitations has drastically declined during the pandemic and has remained low since.

**FIGURE 2:**
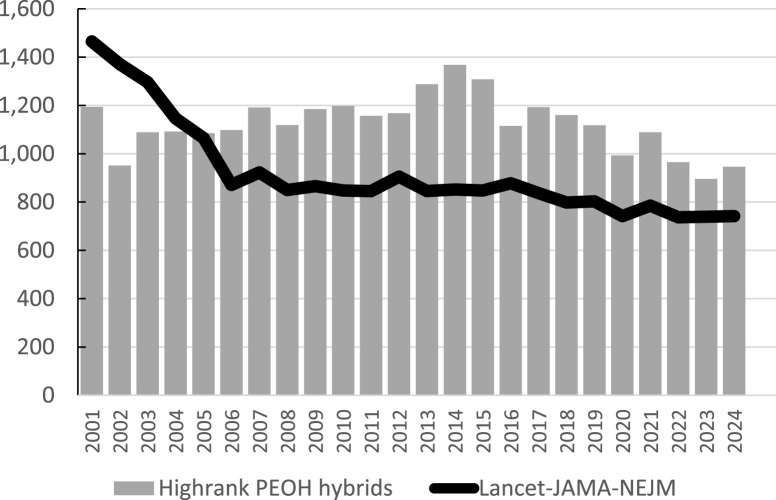
Number of original articles and review articles published in English in seven respected journals *) in the Web-of-Science (WoS) category “Public Environmental and Occupational health” (PEOH) per year (bars) and in Lancet, JAMA and NEJM (dark line), 2001–2024. WoS was accessed on 15 May 2025. As of 2024, all these journals still combine subscriptions with Gold Open Access (hybrid journals). During these years, the seven PEOH journals together with Lancet, JAMA and NEJM were perceived as respected journals of first choice in the science domain of the first author (i.e., environmental epidemiology). All ten journals rank in Q1 of the respective WoS category of the 2024 Journal Impact Factor. None of these quota journals contributed to the accommodation of the steady growth in scientific production shown in Figure 1. *) American Journal of Epidemiology, American Journal of Public Health, Epidemiology, European Journal of Epidemiology, International Journal of Epidemiology, Journal of Epidemiology and Community Health, Journal of Exposure Science and Environmental Epidemiology.

Second, Open Access (OA) journals seem particularly susceptible to problems with finding reviewers given that the natural growth shown in Figure 1 must be accommodated to a large extend by OA journals. It is not in the economic interests of subscription-based journals – now typically under the hybrid model – to steadily increase the number of publications, given that additional publications add costs but not revenues. As shown in Figure 2, many respected journals set very strict annual quota to control costs. The Gold OA model is not jeopardized by growth, since each accepted article also generates revenues. Requests for reviews will therefore over-proportionally increase for OA journals that accommodate this growing demand, while remaining more stable for quota journals. Moreover, quotas also reduce the risk of weakening the journal impact factor (JIF), which tends to decrease as the number of publications – i.e., the denominator in the JIF calculation - increases. As observed in our survey, launched in the context of our Editorial about the peer review crisis [2] (see Supplementary Material), the JIF is the single most important motivator to accept a review invitation.

Thus, paradoxically, finding reviewers may be particularly challenging for medium-ranked OA journals - like IJPH – that do not steer the number of publications with a quota.

Third, the flood of requests for reviews is itself increased by the growing reluctance of scientists to accept review invitations. In the case of IJPH, HE has to approach, on average, 20–40 scientists to secure two reviews, with the tail of the distribution reaching 100. Taken together, the first two arguments ultimately foster the scientists’ perception that they are being spammed by invitations for reviews, sent out by OA journals, including IJPH.

The fourth frustrating strain on the peer review system relates to the rejection cascade, experienced by many scientists on a regular basis: manuscripts ending with a rejection after peer review will in most cases be resubmitted elsewhere, triggering yet another peer review, to ultimately be published by the second if not third chosen journal. This cascade is untransparent and inefficient for the authors and may easily double if not triple the peer review burden on the shoulders of the research community.

The transition to OA confronted IJPH editors with a new challenge, highlighting the importance of the EiC prescreen in managing the peer review crisis. In the previous subscription model, later expanded to hybrid for authors opting for OA, IJPH had an annual publication quota. The prime strategy to fulfill this was to restrict the initiation of peer review during the pre-screen. In the case of IJPH, all pre-screen decisions, including desk rejections, are the sole responsibility of the EiC (see the editorial workflow in Supplementary Figure S1 in the Supplementary Material). This task, rotated on a monthly basis, was coordinated by the Managing Editor, who informed the EiC about the maximum number of submissions to initiate peer review during the respective month. That rate could be as low as 3%–10% of expected submissions, thus forcing the EiC to pick only the most promising submissions. This quota rule camouflaged a challenge now faced by the EiCs: for each and every submission one EiC has to decide whether it is worth sending for peer review or not. This task is rather different from picking only a few top-ranking submissions. Peer reviewing manuscripts with a relatively low chance of success results in higher post-review rejection rates. This, in turn, forces authors to re-submit their revised manuscripts to another journal, subject to a new peer review. Pre-screen decisions should make sure that those advanced to peer review have very good chances to successfully pass the peer review.

Editors of independent journals such as IJPH have little influence on the first three quantitative causes of the peer review crisis. However, the specificity of initiating peer review is fully in their hands. Thus, pre-screening editors have a key role in ameliorating the post-review rejection cascade and the related review burden to be shouldered by the research community. Given the relevance of the specificity of our decisions to initiate peer review, the objective of this study was to better understand, compare and optimize our pre-screen decisions, with the ultimate goal of keeping the post-review rejections reasonably low via a careful steering of the rejections made during the pre-screen, commonly known as a “desk rejection”. The steady increase of the post-review rejection rates of IJPH, observed since the 2021 transition to OA, underscores the need for this investigation.

Though some oppose the editorial strategy of desk rejections [4], general public health sciences journals like IJPH inevitably need this step to be efficient and just to authors, handling editors and reviewers alike. Similar to others [5], a main reason for desk rejections in IJPH is poor fit with the scope of the journal. Flaws in methods and quality coupled with the concerns that the appropriate standard may not be achieved even after peer review also result in desk rejections. Furthermore, limited “innovation,” “originality” or “novelty” provide reasons for desk rejections. However, none of these pre-screen criteria are objectively defined among IJPH editors nor, to the best of our knowledge, in scientific publishing in general. Therefore, the aim of this study was to assess and discuss the agreement of pre-screen decisions among the three EiCs of the IJPH.

## Methods

For several years, the three Co-EiC of IJPH have shared the task of pre-screening equally, taking turns to perform almost all pre-screens for 1 month each. Every year, the monthly assignments are pre-defined, so that each EiC leads 4 months of prescreens in total each year. The prescreen involves an EiC deciding whether or not the manuscript will be further considered by the journal. If the EiC decides that the manuscript has passed the pre-screen, they will request that one of the journal’s AE (with the specific expertise needed for the manuscript) handle the submission by sending it for peer review by external experts. If the manuscript does not pass the prescreen review, it will be immediately rejected by the EiC (Supplementary Figure S1).

For this study, one EiC (A) was in charge for submissions received during November 2024. Our two-stage study was undertaken in the context of the real-world pre-screen tasks and decisions of the first 30 submissions A had to handle between November 1st and 22nd 2024. All stage 1 and stage 2 decisions taken by A were immediately implemented as real-life editorial tasks, so as not to jeopardize the peer review process. The decisions of EiC B and C were blinded “mock” decisions made on the same manuscripts, which contributed to this study only.

For stage 1, A listed all submissions in an excel sheet with six columns: a) a manuscript identifier, b) the date of handling the manuscript, c) an html link to the manuscript on the journal platform, and d) the country of origin of the first author. The country was highlighted given the editorial strategy of IJPH to strengthen the presence of public health sciences from underrepresented regions such as Africa and Latin America. Column e) was used to enter the pre-screen decision (1 = initiation of peer review; 0 = rejected at pre-screen) and f) asked for a short rationale for the decision. In stage 1, decisions of A in column e) and f) were not disclosed to B and C. The latter received the table with links to the 30 manuscripts, without the decisions and rationale of A. EiC B and C independently evaluated the same manuscripts. A, B, and C did not communicate with each other during stage 1.

The purpose of stage 2 was to explore how a “second opinion” would influence the pre-screen decisions. For a few years, where there are self-identified uncertainties about the pre-screen decisions, an EiC occasionally opts for a “second opinion”. The EiC would typically ask the invited HE for pre-screen advice to profit from their more specific expertise in the manuscript in question. This step was part of the real-life pre-screen task of A, who requested a second opinion from the invited HE in four cases. B and C simulated this through a mutual (mock) assessment among the EiCs, rather than with any HE. Specifically, in stage 2, B and C received those manuscripts where the Stage 1 decisions of the EiCs diverged, taken as proxy for uncertainties. The decisions and rationale listed by the three EiCs in stage 1 were disclosed at this stage. Consequently, in stage 2, the EiC had to either defend the original decision or reverse it, if convinced by the opinion of another editor.

In summary, stage 1 decisions of A, B and C were taken independently, without any exchange. In stage 2, A received a second opinion from the chosen HE (real-life peer review), whereas B and C received their second opinions from the other two EiCs. Each EiC then took a final stage 2 decision.

All responses were compiled in the excel sheet for descriptive analyses, including the Pearson correlation comparing A with B and C, as well as B with C. In light of the descriptive nature of this study, we were not interested in estimating true effects, and therefore did not provide confidence intervals or other test statistics.

## Results

Table 1 shows the summary of the decisions, with initial desk rejection rates of 47%, 80% and 60%, for A, B and C, respectively. The final decisions (stage 2) of A included advice from the invited HE of four manuscripts, resulting in three (75%) changes in these (real-live) stage 2 pre-screen decisions. B and C changed their stage 1 decision for 5 of 9 (56%) and 2 of 7 (29%) of the manuscripts re-assessed in stage 2. Second opinions resulted in higher desk rejection rates for A and C while remaining the same in case of B (80%). The stage 2 proportion of manuscripts advanced to peer review was 43%, 20% and 33%, respectively.

**TABLE 1:** Stage 1 decisions of Editor-in-Chief A, B, and C taken for the 30 manuscripts and Stage 2 decisions for re-assessed manuscripts after considering second opinions of handling editors (A) and the other Editors-in-Chief (B,C).

Decisions by editor-in-chief	A	B	C
Stage 1			
0 = reject during pre-screen (desk rejection)	14	24	18
1 = initiation of peer review	16	6	12
*% initiated peer review*	*53%*	*20%*	*40%*
Stage 2			
0 = reject during pre-screen (desk rejection)	17	24	20
1 = Initiation of peer review	13	6	10
*% initiated peer review*	*43%*	*20%*	*33%*
manuscripts re-assessed in stage 2	4	9	7
Prescreen decision change in stage 2	3	5	2
% Stage 2 manuscripts with decision change	75%	56%	29%
% Stage 1 initiated in stage 2	81%	83%	83%

Table 2 summarizes the pattern of agreement among the three EiCs. In stage 1, only 3 (19%) out of 16 manuscripts that passed the pre-screen of A would have been advanced by both, B and C as well. The final real-life pre-screen decision of A (stage 2) initiated peer review for 13 manuscripts, 5 of which (38%) both B and C finally agreed (stage 2) to propose a peer review. A, B and C had full stage 1 agreement (reject or advance) for 43% of the manuscripts, which increased to 67% in stage 2.

**TABLE 2:** Pattern of agreements and disagreements among the pre-screen decisions taken by the three Editors-in-Chief in stage 1 and stage 2, respectively, and correlations of the related decisions (N = 30 manuscripts).

Analyses	Indicators	ST 1 (ABC)	ST 2(ABC)
Correlations	A versus B	0.13	0.57
	A versus C	0.49	0.52
	B Versus C	0.10	0.53
Agreements	ABC initiate review	3	5
	ABC reject in pre-screen (desk rejection)	10	15
	*Total decisions with full agreement (ABC)*	*13*	*20*
	*% of pre-screens with full agreement (ABC)*	*43%*	*67%*
1 Initiates review	A initiates review, BC reject (100)	5	4
	B Initiates review, AC reject (010)	2	0
	C Initiates review, AB reject (001)	2	2
2 Initiate review	A reject, BC initiate review (011)	0	0
	B reject, AC initiate review (101)	7	3
	C reject, AB initiate review (110)	1	1
	Total survey	30	30

The pattern of disagreements shown in Table 2 reveals that papers were more likely to pass the pre-screen of A whereas B had the highest desk rejection rates. Discrepancies decreased in stage 2. In stage 1 decisions, A diverged from the other two with 5 manuscripts, B from the others with 9 and C with 3 manuscripts. In stage 2, A, B and C decisions deviated from the two others for rather similar 4, 3, and 3 manuscripts. As shown by the correlation coefficients, although agreement between the three increased with the second opinion polled in stage 2, it remained at rather modest levels (0.52–0.57).

In seven cases with divergent stage 1 assessments, stage 2 resulted in full agreement among the three editors. Five of those cases ended with a desk rejection whereas two of the seven unanimously passed the stage 2 pre-screen. The editors’ rationale for stage 1 decisions revealed clusters of reasons to reject or to initiate peer review. Poor quality of methods, analyses and/or writing and limited interest for IJPH due to being too small, local, or clinical to be of international public health relevance were equally frequent reasons to reject. Several times, assessors questioned the originality of the study. Explicit arguments for the promotion of peer review were often aligned with editorial strategies of IJPH, such as fostering research from underrepresented regions of the world or societal impact. However, unequal weighting among these soft criteria for pre-screen decisions explained disagreements between the editors to a large extent. Editors expressed uncertainties in stage 1 decisions in relation to these soft dimensions in four of the five manuscripts where editors found agreement to reject in stage 2 only.

## Discussion

The comparison of the pre-screen assessments of the three EiCs showed substantial disagreement about the initiation of peer review. Asking editors for a “second opinion” resulted in higher desk rejection rates and stronger agreement amongst EiCs. Though we cannot formally test whether the specificity of the initiation of peer review did improve thanks to the second opinion, the uncertainties expressed by EiCs in stage 1 in judging soft decision criteria endorse this interpretation. We consider the change from 43% to 67% of agreement after taking a second opinion into account useful and potentially relevant. The 50% increase in the desk-rejections – i.e. 15 manuscripts in stage 2 instead of 10 in stage 1 – is expected to contribute to reducing the rejection cascade. Thus, IJPH editors have now decided to add a “second opinion” to the default workflow if the EiC asks a HE to initiate peer review. Invited HE will now re-evaluate and possibly challenge the initiation of peer review. Whether this will result in more specific decisions and a lower post-review rejection rate awaits to be seen.

Our study is not representative given the rather small sample size of only 30 consecutive submissions and inherent differences among journals in the editorial workflow. Moreover, given that the study had to be embedded in the real submission process, A had to ask other editors – not B or C – to provide a second opinion and to possibly handle the peer review. B and C made instead “mock” decisions in stage 1 and stage 2, without the pressure of the real-life peer review process. Moreover, second opinions in the real-life peer review are provided by the HE, thus, usually a more specific expert in the field of the manuscript as compared to the EiCs. However, we consider it unlikely that these methodological glitches had a relevant impact on the observed results and the broader interpretation.

As mentioned, the prime motivation for our investigations is the peer review crisis. We do not know whether scientists changed their underlying attitudes towards peer review over the past decades. However, the quantitative elements summarized in the introduction are probable causes of an increasing demand for peer review. Unfortunately, editors have no direct influence on most of these general trends. The rejection cascade instead depends to a large extent (if not entirely) on decisions taken by leading editors. Having a strategy at hand – namely increasing the specificity in the promotion to peer review – to lower the post-review rejection rates is promising. If editors of leading journals were to pursue this same goal, the overall initiation of peer review would become more specific, resulting in lower post-review rejection rates. Thus, the peer review burden shouldered by the science community could decrease substantially and provide more space to accommodate the natural growth in the demand for publications. The first revision cycle will usually improve the quality of a paper [6], whereas the added value and quality gain of further peer review cycles organized by other journals decreases with every additional review, until it ultimately vanishes. Thus, the “rejection cascade” is a very inefficient method of quality assurance, which both frustrates authors and causes an additional strain in the science community needed for peer reviews.

Our call for higher specificity in the initiation of peer review and for reductions of the rejection cascade should not be interpreted as an argument to go back to annual quota. Although strict quotas are the easiest way to pick only the very best submissions, they cause a range of additional strains and unwanted conflicts in the publishing system. Though less cumbersome for authors than post-review rejection cascades, desk rejections still add inefficient loops of re-formatting and re-submissions to possibly less selective journals. Quota journals are also a prime cause for launching new journals to accommodate the natural growth. Indeed, according to WoS (accessed 25.5.25), the PEOH category listed 154 journals in 2000 but more than 500 in 2024. Launching new journals in the same fields is not necessarily efficient nor useful but inevitable in a world where quota-journals refuse to accommodate the natural growth. Scientists should indeed question the business model of quota, namely to protect the subscription-based hybrid model and to foster the JIF as one of the correlates of higher revenues. As mentioned, quota journals also put disproportional peer review demands on other journals that agree to accommodate the steady growth of the research production shown in Figure 1. Last but not least, quotas amplify intergenerational inequities. Combining the natural growth in PEOH publications during the past 25 years (shown in Figure 1) with the strict quota endorsed by the leading journals shown in Figure 2, reveals that today’s chances of scientists getting a paper published in these leading journals decreased to one tenth as compared to the year 2000. Fortunately, the IJPH’s transition to OA ended the previous quota requirements, thus editors now have full independence for all decisions to shape the content of IJPH, including the unrestricted right to initiate peer review for all good articles. Inevitably, EiC now instead face the challenge of optimizing the specificity of the initiation of peer review.

Public health sciences are a highly multidisciplinary area, such that the expertise of public health scientists (and the pre-screening EiC) cannot cover the entire field. This is a challenge if pre-screening EiC has to make decisions for manuscripts from relatively unfamiliar sub-fields. This provides a further argument to integrate “second opinions” into the pre-screen decision process of a public health journal in order to reduce the post-peer-review rejection rate. Alternatively, as many large journals do, one could enlarge the number of pre-screening editors to have “section editors” with more specific expertise in each sub-field of public health sciences. However, among larger groups of EiC it will be more difficult to maintain strategic consistency within the journal. Whereas large journals have no other choice, we currently prefer our model of having three EiCs from rather different fields of public health sciences who take full responsibility for all pre-screen and final publishing decisions, supported by an engaged network of associate editors who provide their leading expertise to handle the peer review.

The rationale for the pre-screen decisions provided by A, B and C revealed interesting patterns. As mentioned, disagreements related primarily to “soft criteria” such as “relevance for public health” or values such as “societal impact,” “originality,” “novelty” or “innovation.” The remarkable disagreement observed in our study – even after considering a second opinion - may come as a surprise if not a frustration for researchers. However, we are not aware of any objective operationalization of these criteria, and judgments will therefore inevitably vary among editors, and even within one editor over time. Indeed, criteria such as “novelty” or “relevance” may stay in conflict with editorial strategies of a journal such as societal impact or the wish to foster public health research from regions with the biggest public health challenges and need for local evidence. The latter has been an explicit goal of IJPH for many years. The first authors’ affiliations of the 192 IJPH publications in 2024 are from >50 countries, including 22 from low- and middle-income countries (LMIC). These are less likely to afford to be “the first” or to have the most novel instruments and methodologies to address public health challenges. Where resources are limited, it is crucial to focus public health research on the most pressing rather than the most “novel” issues.

The recent IJPH calls on Aging and Health in Sub-Saharan Africa [7] or Neglected Tropical Diseases During the COVID-19 Pandemic illustrate the editors’ conflicts in weighting soft criteria. The calls did not focus on novelty *per se* but on public health issues of underprivileged regions in the Global South. IJPH editors may also struggle with the soft criterion of “relevance for IJPH readers.” From a Global North perspective, one could easily argue that a paper on dengue fever in a tertiary care hospital in Karachi is too narrow in scope [8]. Moreover, only a limited community among those specialized in dengue research might cite this descriptive local data. Thus, the topic is unlikely to attract large numbers of citations. This exemplifies a further conflict in pre-screen decisions. If quotas and the JIF are given high strategic priority, EiCs will (have to) desk-reject research of rather local relevance while favoring those with a more global perspective. For example, unsurprisingly, a paper about developing a medical education framework for refugee mental health in Switzerland [9], though challenging, possibly “novel” and of “relevance” in the local context, will never attract readers and citations to the same degree as the global update of the WHO Global Air Quality Guidelines’ review about the effects of particulate matter on mortality [10]. The former has not yet (i.e., as of 15.12.25, within 8 months of publishing) attracted citations [9], whereas the latter got more than 10 of its current 40 citations within the first 8 months [10].

It might be tempting to call for agreed upon rating scales or check-lists to assess soft criteria (such as originality, relevance, impact, etc.). However, we believe that for a general public health journal such as IJPH, conflicts between strategic values of global health equity and societal impact versus “originality,” “relevance” or “citations” cannot be resolved with checklists or objective or standardized tools, given how expertise varies among public health scientists. Indeed, what ultimately stands out in a journal dedicated to public health sciences in general - such as IJPH - is the inability of EiC’s to have the same level of expertise across this wide field. This fosters discrepant assessments of “soft criteria.” Thus, adding a “second opinion” prior to initiating peer review may reduce the risk of unbalancing strategic values such as equity in global public health research and societal impact against “originality,” “novelty” or the collection of citations in support of the JIF [11–13].

Highly selective global public health journals may pursue other strategic visions, prioritizing “novelty” and using quotas to foster the JIF, which may compromise quality [14] or balanced representation of authors affiliated with institutions in the global South. For example, the 2024 first authors of the prime journal in global health were primarily affiliated with research institutions in the USA, UK, Switzerland, France and other high income countries (see Supplementary Figure S2 in the Supplementary Material). Indeed, as EiC of IJPH, we are aware of the conflicts between promoting research from institutions in regions with the biggest public health challenges and needs for societal impact versus favoring “novelty,” “originality” or “innovation” alone, which is often the privilege of resourceful research institutions in the global North.

Our call for lower post-review rejection rates may be provocative and counterintuitive for many researchers, editors and academic assessors. Indeed, despite the lack of any objective evidence, many might share narratives that feature high rejection rates as indicators of “quality.” However, the narrative should be challenged. High rejection rates should not be seen as a proxy for quality but as another ill-defined incentive given that journals could rather easily manipulate post-review rejection rates by initiating peer reviews for low quality manuscripts. Fraudulent journals will find ways to get high numbers of irrelevant or fake paper submissions to demonstrate high rejection rates in collaboration with circles who allow reviews to be written by artificial intelligence [15]. Instead, we propose that editors question the traditional pride in showcasing “high rejection rates,” which may correlate poorly with quality. Instead, high specificity in the initiation of peer review, and lower rejection rates after peer review, should become an agreed upon target and source of pride. Science and the research community do not need inefficient rejection cascades after peer review, but strong pre-screen decisions by editors.

A relevant step toward the abatement of the rejection cascade via high specificity in the initiation of peer review and low post-review rejection rates would be the transparent disclosure of the pre- and post-review rejection rates. Unfortunately, journals have rarely communicated those distinct rates. As mentioned, we have observed a steady increase in the post-review rejection rates since the 2021 transition to OA, which was paralleled by the pandemic. Rejections after peer review were below 20% in 2021 but have since passed one third of all peer-reviewed manuscripts. We asked the EiC of three leading PEOH journals about these rates. Whereas two society journals provided at least partial information, confirming similar situations with some room for lowering post-review rejection rates, the selected for-profit journal refused to provide any insights.

Unneeded requests for peer review as caused by the rejection cascade could also be challenged if the submission/rejection history of published articles had to be disclosed in the final publication, including all previous submission dates and journals. Publishing these indicators in an openly accessible data base such as Clarivate’s Web of Science would reveal the contribution of desk-rejections and rejection cascades after peer review to the strain caused on the researchers. Last but not least, adding all former reviews to re-submissions of manuscripts to other journals should become a required default instead of the current situation where this is, at best, an optional service provided in case authors re-submit to a journal of the same publisher.

Our proposal to base the initiation of peer review on the assessment of two editors might raise questions about efficiency of the journal performance. Whether our model is less efficient or not may depend on the workflows and functionalities of the journals’ platform. In our case, efficiency is not affected. The pre-screening EiC invites a HE to initiate the peer review as before. However, this invitation comes with the explicit request to (possibly) question the initiation of peer review. If the HE agrees to start the review, procedures are as before (see Supplementary Figure S1). If not, the HE declines the initiation with one click, sending a short rationale to the EiC. In case the latter agrees, desk-rejection is the next fast and final step. So far, our experience shows that it is extremely rare that the EiC opens a discussion with the HE to ”enforce” a peer review. Thus, the adapted workflow (also shown in Supplementary Figure S1) results in most cases in a substantial reduction of the HE’s workload (and by extension that of the reviewers who were not invited). Our experience of increased efficiency may not be generalizable to much larger journals where anonymity and competition among large pools of editors may jeopardize the sense of trust and respect of each other’s competences and expertise currently prevailing among the editors of IJPH. We abstain from promoting a second opinion in case the EiC prefers a desk rejection. These are far too numerous (some 70%–90% of all submissions) to involve a second editor by default, since such change in the workflow would result in a substantial loss of time and editorial efficiency.

Our study did not directly assess the specificity of the initiation of peer review nor the sensitivity of the desk rejections, as we do not yet have full information of the fate of all 30 manuscripts in the study. Both indicators matter for the assessment and improvement of peer review efficiency. So far (i.e., as of 14.12.2025) 21% of the manuscripts with peer review were finally rejected (i.e. 3 out of 14). More importantly, five of all rejected manuscripts of our study are now published in other journals, including four desk rejections and one after peer review. The latter of these along with two desk rejections for which EiC A questioned the added value were published in journals with a higher JIF than IJPH. Those three papers already collected twelve citations in total (14.12.25). Only one desk-rejection was due to poor fit to the mission of IJPH. At most one of the three EiC proposed initiation of a peer review for these five manuscripts. Though we do not yet know the fate of 14 rejections, these preliminary investigations confirm that peer review decisions are not only based on standardized criteria, but also on a range of soft criteria.

To better understand both the specificity and sensitivity of the pre-screen decisions and the related quality of published articles, we would need a larger long-term study that follows the ultimate fate and publication success of submissions rejected at pre-screen and accepted or rejected after peer review, with and without a “second opinion”. Such studies could foster quality-oriented criteria to evaluate decisions of editors and thus the quality of a journal. Such quality-based criteria could replace biased subjective academic policies which too often acknowledge only the journal impact factor and/or lack evidence [16, 17].

### Conclusion

Our study demonstrates disagreement between the pre-screen decisions of three EiCs, which were reduced after considering a “second opinion” of a proposed promotion to peer review. This confirms that the prime criteria guiding pre-screen decisions, such as novelty, originality and relevance, are not objectively defined indicators but decisions taken on a continuous scale. These may in turn even stay in conflict with values of high relevance for global and public health sciences such as societal impact. Taking a second opinion into account resulted in higher desk rejection rates. Thus, a second opinion may not only harmonize pre-screen decisions but hopefully result in more specific decisions to initiate peer review and, thus, lower rejection rates after peer-review. The latter would be a highly welcome development to reduce the “rejection cascade” in public health sciences, eliminating the unproductive part of the strain that the peer review puts on the shoulders of the research community. This study also confirms a message of particular relevance to scientists frustrated by pre-screen rejections: do not give up! These fast decisions are inevitably a complex mixture of objective and subjective soft criteria. Thus, an element of “chance” may also be present.
