# Supplementary material for: Desk Rejection Decisions – Do Co-Editors-In-Chief of This Journal Agree?
Source: Int J Public Health. 2026 Feb 26;71:1608909. doi: 10.3389/ijph.2026.1608909 (PMC12979234; doi:10.3389/ijph.2026.1608909)
Supplement: Supplementary file 1 [file Table1.docx]

**ONLINE SUPPLEMENT RELATED TO:**

**Desk rejection decisions – do Co-Editors-in-Chief of this journal agree?**

Nino Künzli 1,2,3 Olaf von dem Knesebeck 4, Andrea Madarasova Geckova 5, Sunghea Park 2,3, Christopher Woodrow 1,2,3

1 Swiss School of Public Health (SSPH+), Zurich, Switzerland

2 Swiss Tropical and Public Health Institute, Allschwil, Switzerland

3 University of Basel, Basel Switzerland.

4 Institute of Medical Sociology, University of Hamburg, Germany

5 Department of Health Psychology and Research Methodology, Pavol Jozef Šafárik University in Košice, Slovakia

**Details about the survey regarding incentives to accept invitations to review a manuscript**

The Editorial (see Reference 1) about the peer review crisis encouraged readers to participate in a short survey about their engagement in and attitudes toward peer review. Section 3 of the survey asked the following question:

How do the following features of journals influence your decision to accept or to decline an invitation to review?

The response scale ranged from 0 to 10, meaning “0: More likely to decline, 5: Neutral, 10: More likely to accept”. The following nine features were listed for the assessment:

- 1. Journal is a “society journal” (i.e. not owned by a company)
  2. Business model of the journal is “not-for–profit”
  3. Journal has a high impact factor
  4. Journal is fully Open Access
  5. Journal waives publishing fee (APC) for authors from low and middle income countries
  6. Journal is Open Access but charges no APC (platinum open access)
  7. Journal is published by a traditional publisher company (such as SpringerNature, Elsevier etc.)
  8. Journal is published by a new(er) Open Access publisher (such as MDPI, Frontiers etc.)
  9. Journal is published open access by a pre-print platform with post-publication peer review

Defining responses 0-4 for as “Disagree”, 5 for “Neutral” and 6-10 for “Agree” to review, results revealed that a high journal impact factor motivated 80% of the 599 respondents to accept an invitation to review. A not-for-profit business model motivated 67% to engage in a review. Features a. and d. to g. were perceived as motivators among 50-60% whereas respondents were much more hesitant to review for journals of new(er) OA publishers (33%) or for pre-print platforms (22%). Results look very similar with different categorizations such as considering e.g. only responses 8 to 10 as “agree”.

Reference:

1. Künzli N, Berger A, Czabanowska K, Lucas R, Madarasova Geckova A, Mantwill S, von dem Knesebeck O. «I Do Not Have Time»-Is This the End of Peer Review in Public Health Sciences? Public Health Reviews (2022) 17:43. doi: 10.3389/phrs.2022.1605407.

**Figure S1**: The figure visualizes the traditional workflows of the SSPH+ Journals, International Journal of Public Health and Public Health Reviews and the now adapted workflow. In the latter, the EiC still invites a Handling Editor (HE) to initiate the peer review. The HE is, however, explicitly asked for a second opinion whether or not to initiate a peer review. In case the HE would rather not, and the EiC agrees, the latter will make the desk rejection. All other steps remain the same. Thus, usual desk rejections remain the sole task and decision of the EiC.

**Figure S2:** Prime affiliation country of the first authors of original articles or reviews published in 2024 by Lancet Global Health. The figure is based on the Clarivate Web of Science data base, accessed on 31.5.2025, with the following WoS search: <https://www.webofscience.com/wos/woscc/summary/e23a490f-1215-4989-876e-9a59b1cc7587-0165bb6733/relevance/1>
